# Longitudinal Changes in Cognitive Function, Muscle Mass, and Phase Angle Among Older Adults with Alzheimer disease: a Two‐year Longitudinal Study

**DOI:** 10.1002/alz70857_102334

**Published:** 2025-12-25

**Authors:** Derong Zeng, Mu‐Hsing Ho, Teruaki Kawasaki, Ichiro Akiguchi, Ayae Kinoshita

**Affiliations:** ^1^ kyoto university, kyoto, kyoto, Japan; ^2^ The University of Hong Kong, Hongkong, Hongkong, China; ^3^ Kyoto Clinical and Translational Research Center for Neurocognitive Disorders, Kyoto, Japan; ^4^ Kyoto Clinical and Translational Research Center for Neurocognitive Disorders, kyoto, kyoto, Japan; ^5^ Kyoto University Graduate School of Medicine, Kyoto, Japan

## Abstract

**Background:**

We previously reported an association between higher phase angle (PhA) and better cognitive performance in older adults with cognitive impairment^1)^. However, whether PhA changes over time in relation to cognitive decline remains unclear.

**Methods:**

Between 2018 and 2022, we recruited 240 older adults (mean age 82.4 ± 6.5 years; mean MMSE 21.9 ± 5.0) who had objective or subjective cognitive impairments over two consecutive years and completed both MMSE and physical assessments. Cognitive function was measured using the Mini‐Mental State Examination (MMSE). Physical assessments included body mass index, total muscle mass, limb skeletal muscle mass, fat percentage, estimated bone mass, and PhA. Linear mixed‐effects models examined changes from the first to second year, adjusting for age, sex, education, and height. One‐year change scores were calculated for all measures, and comparisons were made between participants with Alzheimer disease (AD) and those with mild cognitive impairment (MCI).

**Results:**

MMSE scores declined significantly from year one to year two (*p* < 0.001), as did total and limb skeletal muscle mass (both *p* < 0.05) and PhA (*p* < 0.001). Other anthropometric measures (body mass index, fat percentage, visceral fat, and estimated bone mass) remained stable. At baseline, participants with AD had lower MMSE scores (mean difference 7.31, *p* < 0.001); however, their one‐year decline (–1.01 points) did not differ significantly from that of MCI participants (–1.68 points, *p* =  0.061). In multiple regression analyses, lower education and AD status significantly predicted greater MMSE decline (*p* < 0.05).

**Conclusions:**

Over one year, older adults at risk for cognitive decline showed decreases in MMSE, muscle mass, and PhA, while other anthropometric measures remained unchanged. Although AD participants consistently had lower cognitive scores, their rate of decline was similar to those with MCI. Monitoring both physical and cognitive indicators, particularly PhA, may support early detection of deterioration in older adults with or at risk for AD.

**References**

Zeng, D., Nishimoto, H., Kawasaki, T., Akiguchi, I., Fukui, K., & Kinoshita, A. (2025). Association of cognitive function and phase angle in older adults with cognitive impairment. Archives of Gerontology and Geriatrics Plus, 2(1), 100122. https://doi.org/10.1016/j.aggp.2025.100122.

